# Supplementary material for: Iterative improvement in the automatic modular design of robot swarms
Source: PeerJ Comput Sci. 2020 Dec 7;6:e322. doi: 10.7717/peerj-cs.322 (PMC7924708; doi:10.7717/peerj-cs.322)
Supplement: Supplemental Information 3 [file peerj-cs-06-322-s003.zip › argos3/doc/api/standalone/a00560_source.html]

ARGoS: plugins/robots/spiri/simulator/qtopengl\_spiri.cpp Source File


- Main Page
- Related Pages
- Namespaces
- Classes
- Files

- File List
- File Members

# plugins/robots/spiri/simulator/qtopengl\_spiri.cpp

Go to the documentation of this file.

```
00001 
00007 #include "qtopengl_spiri.h"
00008 #include "spiri_entity.h"
00009 #include <argos3/core/simulator/entity/embodied_entity.h>
00010 #include <argos3/core/utility/math/vector2.h>
00011 #include <argos3/core/utility/math/vector3.h>
00012 #include <argos3/plugins/simulator/entities/led_equipped_entity.h>
00013 #include <argos3/plugins/simulator/visualizations/qt-opengl/qtopengl_widget.h>
00014 
00015 namespace argos {
00016 
00017    /****************************************/
00018    /****************************************/
00019 
00020    /* All measures are in meters */
00021    static const GLfloat SIDE        = 0.470f;
00022    static const GLfloat DIAGONAL    = SIDE * ::sqrt(2);
00023    static const GLfloat OUT_RADIUS  = DIAGONAL / 5.0f;
00024    static const GLfloat IN_RADIUS   = OUT_RADIUS * 0.95f;
00025    static const GLfloat HEIGHT      = 0.090f;
00026 
00027    static const GLfloat PROP_HEIGHT    = HEIGHT / 2.0f;
00028    static const GLfloat PROP_ELEVATION = HEIGHT - PROP_HEIGHT;
00029    static const GLfloat PROP_ARM       = DIAGONAL / 2.0f - OUT_RADIUS;
00030    
00031    static const GLfloat LEG_RADIUS = OUT_RADIUS * 0.05f;
00032    static const GLfloat LEG_HEIGHT = HEIGHT - PROP_HEIGHT;
00033 
00034    static const GLfloat BODY_SIDE      = OUT_RADIUS;
00035    static const GLfloat BODY_HEIGHT    = HEIGHT * 3.0f / 4.0f;
00036    static const GLfloat BODY_ELEVATION = HEIGHT - BODY_HEIGHT;
00037 
00038    /****************************************/
00039    /****************************************/
00040 
00041    CQTOpenGLSpiri::CQTOpenGLSpiri() :
00042       m_unVertices(40) {
00043       /* Reserve the needed display lists */
00044       m_unList = glGenLists(1);
00045       glNewList(m_unList, GL_COMPILE);
00046       MakeModel();
00047       glEndList();
00048    }
00049 
00050    /****************************************/
00051    /****************************************/
00052 
00053    CQTOpenGLSpiri::~CQTOpenGLSpiri() {
00054       glDeleteLists(m_unList, 1);
00055    }
00056 
00057    /****************************************/
00058    /****************************************/
00059 
00060    void CQTOpenGLSpiri::Draw(CSpiriEntity& c_entity) {
00061       glCallList(m_unList);
00062       /* Save attributes and current matrix */
00063       glPushAttrib(GL_LINE_BIT);
00064       /* Set line attributes */
00065       glEnable(GL_LINE_SMOOTH);
00066       glLineWidth(2.0);
00067       /* Set color */
00068       SetMainBodyMaterial();
00069       /* Draw ray */
00070       glBegin(GL_LINES);
00071       glVertex3f(0,0,0);
00072       glVertex3f(DIAGONAL,0,0);
00073       glEnd();
00074       /* Restore saved stuff */
00075       glPopAttrib();
00076    }
00077 
00078    /****************************************/
00079    /****************************************/
00080 
00081    void CQTOpenGLSpiri::SetPlasticMaterial() {
00082       const GLfloat pfColor[]     = {   1.0f, 1.0f, 1.0f, 1.0f };
00083       const GLfloat pfSpecular[]  = {   0.9f, 0.9f, 0.9f, 1.0f };
00084       const GLfloat pfShininess[] = { 100.0f                   };
00085       const GLfloat pfEmission[]  = {   0.0f, 0.0f, 0.0f, 1.0f };
00086       glMaterialfv(GL_FRONT_AND_BACK, GL_AMBIENT_AND_DIFFUSE, pfColor);
00087       glMaterialfv(GL_FRONT_AND_BACK, GL_SPECULAR,            pfSpecular);
00088       glMaterialfv(GL_FRONT_AND_BACK, GL_SHININESS,           pfShininess);
00089       glMaterialfv(GL_FRONT_AND_BACK, GL_EMISSION,            pfEmission);
00090    }
00091 
00092    /****************************************/
00093    /****************************************/
00094 
00095    void CQTOpenGLSpiri::SetPropellerMaterial() {
00096       const GLfloat pfColor[]     = {   0.7f, 0.7f, 0.7f, 1.0f };
00097       const GLfloat pfSpecular[]  = {   0.9f, 0.9f, 0.9f, 1.0f };
00098       const GLfloat pfShininess[] = { 100.0f                   };
00099       const GLfloat pfEmission[]  = {   0.0f, 0.0f, 0.0f, 1.0f };
00100       glMaterialfv(GL_FRONT_AND_BACK, GL_AMBIENT_AND_DIFFUSE, pfColor);
00101       glMaterialfv(GL_FRONT_AND_BACK, GL_SPECULAR,            pfSpecular);
00102       glMaterialfv(GL_FRONT_AND_BACK, GL_SHININESS,           pfShininess);
00103       glMaterialfv(GL_FRONT_AND_BACK, GL_EMISSION,            pfEmission);
00104    }
00105 
00106    /****************************************/
00107    /****************************************/
00108 
00109    void CQTOpenGLSpiri::SetMainBodyMaterial() {
00110       const GLfloat pfColor[]     = {   0.0f, 0.0f, 1.0f, 1.0f };
00111       const GLfloat pfSpecular[]  = {   0.9f, 0.9f, 0.9f, 1.0f };
00112       const GLfloat pfShininess[] = { 100.0f                   };
00113       const GLfloat pfEmission[]  = {   0.0f, 0.0f, 0.0f, 1.0f };
00114       glMaterialfv(GL_FRONT_AND_BACK, GL_AMBIENT_AND_DIFFUSE, pfColor);
00115       glMaterialfv(GL_FRONT_AND_BACK, GL_SPECULAR,            pfSpecular);
00116       glMaterialfv(GL_FRONT_AND_BACK, GL_SHININESS,           pfShininess);
00117       glMaterialfv(GL_FRONT_AND_BACK, GL_EMISSION,            pfEmission);
00118    }
00119 
00120    /****************************************/
00121    /****************************************/
00122 
00123    void CQTOpenGLSpiri::MakeModel() {
00124       glPushMatrix();
00125       glRotatef(45.0f, 0.0f, 0.0f, 1.0f);
00126       /* Main body */
00127       MakeMainBody();
00128       /* Front propeller */
00129       glTranslatef(PROP_ARM, 0.0f, 0.0f);
00130       MakePropeller();
00131       /* Left propeller */
00132       glTranslatef(-PROP_ARM, PROP_ARM, 0.0f);
00133       MakePropeller();
00134       /* Back propeller */
00135       glTranslatef(-PROP_ARM, -PROP_ARM, 0.0f);
00136       MakePropeller();
00137       /* Right propeller */
00138       glTranslatef(PROP_ARM, -PROP_ARM, 0.0f);
00139       MakePropeller();
00140       glTranslatef(0.0f, PROP_ARM, 0.0f);
00141       glPopMatrix();
00142    }
00143 
00144    /****************************************/
00145    /****************************************/
00146 
00147    void CQTOpenGLSpiri::MakePropeller() {
00148       glPushMatrix();
00149       SetPlasticMaterial();
00150       /*
00151        * Leg
00152        */
00153       MakeCylinder(LEG_RADIUS, LEG_HEIGHT);
00154       /*
00155        * Propeller itself
00156        */
00157       /* Go above the leg */
00158       glTranslatef(0.0f, 0.0f, PROP_ELEVATION);
00159       /* Draw propeller side surfaces */
00160       MakeCylinderSurface(OUT_RADIUS, PROP_HEIGHT);
00161       glCullFace(GL_FRONT);
00162       MakeCylinderSurface(IN_RADIUS, PROP_HEIGHT);
00163       glCullFace(GL_BACK);
00164       /* Draw propeller bottom */
00165       glRotatef(180.0f, 1.0f, 0.0f, 0.0f);
00166       SetPlasticMaterial();
00167       MakeRing(OUT_RADIUS, IN_RADIUS);
00168       /* Draw rotating propeller */
00169       glRotatef(-180.0f, 1.0f, 0.0f, 0.0f);
00170       glTranslatef(0.0f, 0.0f, PROP_HEIGHT / 2.0f);
00171       SetPropellerMaterial();
00172       MakeDisk(IN_RADIUS);
00173       /* Draw propeller top */
00174       glTranslatef(0.0f, 0.0f, PROP_HEIGHT / 2.0f);
00175       SetPlasticMaterial();
00176       MakeRing(OUT_RADIUS, IN_RADIUS);
00177       glPopMatrix();
00178    }
00179 
00180    /****************************************/
00181    /****************************************/
00182 
00183    void CQTOpenGLSpiri::MakeMainBody() {
00184       glPushMatrix();
00185       SetMainBodyMaterial();
00186       glTranslatef(0.0f, 0.0f, BODY_ELEVATION);
00187       MakeBox(BODY_SIDE, BODY_SIDE, BODY_HEIGHT);
00188       glPopMatrix();
00189    }
00190 
00191    /****************************************/
00192    /****************************************/
00193 
00194    void CQTOpenGLSpiri::MakeRing(GLfloat f_out_radius,
00195                                  GLfloat f_in_radius) {
00196       CVector2 cVertex(1.0f, 0.0f);
00197       CRadians cAngle(CRadians::TWO_PI / m_unVertices);
00198       glBegin(GL_QUAD_STRIP);
00199       glNormal3f(0.0f, 0.0f, 1.0f);
00200       for(GLuint i = 0; i <= m_unVertices; i++) {
00201          glVertex3f(cVertex.GetX() * f_in_radius, cVertex.GetY() * f_in_radius, 0.0f);
00202          glVertex3f(cVertex.GetX() * f_out_radius, cVertex.GetY() * f_out_radius, 0.0f);
00203          cVertex.Rotate(cAngle);
00204       }
00205       glEnd();      
00206    }
00207 
00208    /****************************************/
00209    /****************************************/
00210 
00211    void CQTOpenGLSpiri::MakeDisk(GLfloat f_radius) {
00212       CVector2 cVertex(f_radius, 0.0f);
00213       CRadians cAngle(CRadians::TWO_PI / m_unVertices);
00214       glBegin(GL_POLYGON);
00215       glNormal3f(0.0f, 0.0f, 1.0f);
00216       for(GLuint i = 0; i <= m_unVertices; i++) {
00217          glVertex3f(cVertex.GetX(), cVertex.GetY(), 0.0f);
00218          cVertex.Rotate(cAngle);
00219       }
00220       glEnd();
00221    }
00222 
00223    /****************************************/
00224    /****************************************/
00225 
00226    void CQTOpenGLSpiri::MakeCylinderSurface(GLfloat f_radius,
00227                                             GLfloat f_height) {
00228       CVector2 cVertex(f_radius, 0.0f);
00229       CRadians cAngle(CRadians::TWO_PI / m_unVertices);
00230       glEnable(GL_NORMALIZE);
00231       glBegin(GL_QUAD_STRIP);
00232       for(GLuint i = 0; i <= m_unVertices; i++) {
00233          glNormal3f(cVertex.GetX(), cVertex.GetY(), 0.0f);
00234          glVertex3f(cVertex.GetX(), cVertex.GetY(), f_height);
00235          glVertex3f(cVertex.GetX(), cVertex.GetY(), 0.0f);
00236          cVertex.Rotate(cAngle);
00237       }
00238       glEnd();
00239       glDisable(GL_NORMALIZE);
00240    }
00241 
00242    /****************************************/
00243    /****************************************/
00244 
00245    void CQTOpenGLSpiri::MakeCylinder(GLfloat f_radius,
00246                                      GLfloat f_height) {
00247       glPushMatrix();
00248       MakeDisk(f_radius);
00249       MakeCylinderSurface(f_radius, f_height);
00250       glRotatef(180.0f, 1.0f, 0.0f, 0.0f);
00251       MakeDisk(f_radius);
00252       glPopMatrix();
00253    }
00254 
00255    /****************************************/
00256    /****************************************/
00257 
00258    void CQTOpenGLSpiri::MakeBox(GLfloat f_length,
00259                                 GLfloat f_width,
00260                                 GLfloat f_height) {
00261       glEnable(GL_NORMALIZE);
00262       glPushMatrix();
00263       glScalef(f_length, f_width, f_height);
00264       /* This part covers the top and bottom faces (parallel to XY) */
00265       glBegin(GL_QUADS);
00266       /* Bottom face */
00267       glNormal3f(0.0f, 0.0f, -1.0f);
00268       glVertex3f( 0.5f,  0.5f, 0.0f);
00269       glVertex3f( 0.5f, -0.5f, 0.0f);
00270       glVertex3f(-0.5f, -0.5f, 0.0f);
00271       glVertex3f(-0.5f,  0.5f, 0.0f);
00272       /* Top face */
00273       glNormal3f(0.0f, 0.0f, 1.0f);
00274       glVertex3f(-0.5f, -0.5f, 1.0f);
00275       glVertex3f( 0.5f, -0.5f, 1.0f);
00276       glVertex3f( 0.5f,  0.5f, 1.0f);
00277       glVertex3f(-0.5f,  0.5f, 1.0f);
00278       glEnd();
00279       /* This part covers the faces (South, East, North, West) */
00280       glBegin(GL_QUADS);
00281       /* South face */
00282       glNormal3f(0.0f, -1.0f, 0.0f);
00283       glVertex3f(-0.5f, -0.5f, 1.0f);
00284       glVertex3f(-0.5f, -0.5f, 0.0f);
00285       glVertex3f( 0.5f, -0.5f, 0.0f);
00286       glVertex3f( 0.5f, -0.5f, 1.0f);
00287       /* East face */
00288       glNormal3f(1.0f, 0.0f, 0.0f);
00289       glVertex3f( 0.5f, -0.5f, 1.0f);
00290       glVertex3f( 0.5f, -0.5f, 0.0f);
00291       glVertex3f( 0.5f,  0.5f, 0.0f);
00292       glVertex3f( 0.5f,  0.5f, 1.0f);
00293       /* North face */
00294       glNormal3f(0.0f, 1.0f, 0.0f);
00295       glVertex3f( 0.5f,  0.5f, 1.0f);
00296       glVertex3f( 0.5f,  0.5f, 0.0f);
00297       glVertex3f(-0.5f,  0.5f, 0.0f);
00298       glVertex3f(-0.5f,  0.5f, 1.0f);
00299       /* West face */
00300       glNormal3f(-1.0f, 0.0f, 0.0f);
00301       glVertex3f(-0.5f,  0.5f, 1.0f);
00302       glVertex3f(-0.5f,  0.5f, 0.0f);
00303       glVertex3f(-0.5f, -0.5f, 0.0f);
00304       glVertex3f(-0.5f, -0.5f, 1.0f);
00305       glEnd();
00306       /* The shape definitions is finished */
00307       glPopMatrix();
00308       glDisable(GL_NORMALIZE);
00309    }
00310 
00311    /****************************************/
00312    /****************************************/
00313 
00314    class CQTOpenGLOperationDrawSpiriNormal : public CQTOpenGLOperationDrawNormal {
00315    public:
00316       void ApplyTo(CQTOpenGLWidget& c_visualization,
00317                    CSpiriEntity& c_entity) {
00318          static CQTOpenGLSpiri m_cModel;
00319          c_visualization.DrawRays(c_entity.GetControllableEntity());
00320          c_visualization.DrawEntity(c_entity.GetEmbodiedEntity());
00321          m_cModel.Draw(c_entity);
00322       }
00323    };
00324 
00325    class CQTOpenGLOperationDrawSpiriSelected : public CQTOpenGLOperationDrawSelected {
00326    public:
00327       void ApplyTo(CQTOpenGLWidget& c_visualization,
00328                    CSpiriEntity& c_entity) {
00329          c_visualization.DrawBoundingBox(c_entity.GetEmbodiedEntity());
00330       }
00331    };
00332 
00333    REGISTER_QTOPENGL_ENTITY_OPERATION(CQTOpenGLOperationDrawNormal, CQTOpenGLOperationDrawSpiriNormal, CSpiriEntity);
00334 
00335    REGISTER_QTOPENGL_ENTITY_OPERATION(CQTOpenGLOperationDrawSelected, CQTOpenGLOperationDrawSpiriSelected, CSpiriEntity);
00336 
00337    /****************************************/
00338    /****************************************/
00339 
00340 }
```

---

Generated on 10 Jul 2018 for ARGoS by 
 1.6.1
